# Development of a Multiplex PCR and Magnetic DNA Capture Assay for Detecting Six Species Pathogens of the Genera *Anaplasma* and *Ehrlichia* in Canine, Bovine, Caprine and Ovine Blood Samples from Grenada, West Indies

**DOI:** 10.3390/pathogens10020192

**Published:** 2021-02-10

**Authors:** Bhumika Sharma, Roman R. Ganta, Diana Stone, Andy Alhassan, Marta Lanza-Perea, Vanessa Matthew Belmar, Inga Karasek, Elizabeth Cooksey, Catherine M. Butler, Kathryn Gibson, Melinda J. Wilkerson

**Affiliations:** 1 Department of Pathobiology, School of Veterinary Medicine, St. George’s University, St. George, West Indies, Grenada; dstone@sgu.edu (D.S.); aalhass1@sgu.edu (A.A.); Vmatthew@sgu.edu (V.M.B.); kathryn.gibson@usda.gov (K.G.); mwilkers@sgu.edu (M.J.W.); 2 Center of Excellence of Vector Borne Diseases, Department of Diagnostic Medicine/Pathobiology, College of Veterinary Medicine, Kansas State University, Manhattan, KS 66506, USA; rganta@vet.k-state.edu; 3 Department of Small Animal Medicine & Surgery, School of Veterinary Medicine, St Georges University, St. George, West Indies, Grenada; Mperea@sgu.edu; 4 Department of Large Animal Medicine & Surgery, School of Veterinary Medicine, St. George’s University, St. George, West Indies, Grenada; ikarasek1@sgu.edu (I.K.); Ecooksey@sgu.edu (E.C.); cwerners@sgu.edu (C.M.B.)

**Keywords:** *Anaplasma*, *Ehrlichia*, PCR, xMAP

## Abstract

Infections with tick-borne pathogens belonging to *Anaplasma/Ehrlichia* in various vertebrate hosts are a persistent problem resulting in nonspecific clinical signs during early infection. Diagnosis of single and multi-infections with these pathogens, causing diseases in companion/agricultural animals and people, remains a challenge. Traditional methods of diagnosis, such as microscopy and serology, have low sensitivity and specificity. Polymerase chain reaction (PCR) assays are widely used to detect early-phase infections, since these have high sensitivity and specificity. We report the development and validation of an assay involving PCR followed by magnetic capture method using species-specific oligonucleotides to detect six *Anaplasma/Ehrlichia* species pathogens in canine, bovine, caprine, and ovine blood samples. Overall, the assay application to 455 samples detected 30.1% (137/455) positives for one or more out of six screened pathogens. Single-pathogen infections were observed in 94.9% (130/137) of the positive samples, while co-infections were detected in 5.1% (7/137). *Anaplasma marginale* infection in cattle had the highest detection rate (34.4%), followed by canines positive for *Anaplasma platys* (16.4%) and *Ehrlichia canis* (13.9%). The assay aided in documenting the first molecular evidence for *A. marginale* in cattle and small ruminants and *Ehrlichia chaffeensis* and *Ehrlichia ewingii* in dogs in the Caribbean island of Grenada.

## 1. Introduction

For over three decades, *Anaplasma* and *Ehrlichia* species pathogens have been known to cause diseases in humans, while in pets and livestock these infections have been well-documented for many decades [1,2,3]. In the United States, human infections with *Anaplasma* and *Ehrlichia* species are identified as the second leading cause of tick-borne diseases after Lyme disease [4]. Clinical outcomes of ehrlichiosis and anaplasmosis vary from asymptomatic infections to severe, potentially fatal illness in animals and humans. Two or more tick-borne infections are also common in vertebrate hosts [5,6,7,8,9,10,11,12,13,14,15,16,17]. Multi-infections with tick-borne pathogens may enhance the disease severity and complicate the clinical presentation in a host [18,19,20]. Gaunt et al. [21] reported a greater pathophysiological response in dogs experimentally co-infected with *Ehrlichia canis* and *Anaplasma platys*, than when infected with either one of the pathogens. Multiple-pathogen infections can also persist for months to years and complicate a patient’s clinical presentation, substantially influencing the progression of the diseases, while also creating challenges for laboratory diagnosis [13,22,23]. 

Early detection of these infections, when antibiotic treatment is most effective, is often very challenging. This is because early signs and symptoms of these illnesses are nonspecific, making clinical diagnosis difficult [24]. Assays that can rapidly confirm and discriminate between tick-borne rickettsial pathogens are limited and not readily available at an affordable cost. The Indirect immunofluorescence antibody (IFA) assays performed on paired acute and convalescent sera are considered the gold-standard for serologic confirmation of rickettsial infections [25,26]. However, IFA assays are insensitive during the acute phase of rickettsial infection [24,27,28,29,30], because during the early infection stage pathogen-specific antibodies are yet to develop. For tick-borne rickettsial infections, seroconversion usually occurs within two to four weeks, at which time pathogen-specific antibodies can be detected. Therefore, the Centers for Disease Prevention and Control, USA, recommends performing an IgG IFA assay on acute and convalescent-phase samples (sampled two to four weeks apart) in tandem, a four-fold or greater increase in the antibody titer is evidence of seroconversion and reflects current infection [30]. For the detection of *Anaplasma marginale* in particular, the World Organization for Animal Health recommends performing microscopic examination of the freshly prepared blood smears as well as polymerase chain reaction (PCR) assays [31]. PCR assays are based on the principle of artificial amplification of species-specific DNA and are widely used for rapid, sensitive, and specific detection of *Anaplasma/Ehrlichia* species, in the whole blood specimens collected during the acute stages of illness. These molecular methods include both conventional and real-time quantitative PCR assays targeting mostly *16S rRNA* or *16S rDNA* [6,32,33,34,35,36,37]. Other PCR assays use primers targeting genes such as *dsb* [38], *groEL* [39,40], *msp1a*, and *msp4* of *A. marginale* [41,42], major outer membrane protein genes of *Ehrlichia* species such as *p28-p30/MAP1* [43,44], and citrate synthase gene *gltA* [45]. However, most of these assays only detect a limited number of *Anaplasma/Ehrlichia* species. Recently, new technologies have been developed for molecular diagnosis of tick-borne rickettsial infections and identifying the infecting agent. For example, Michelet et al., [46] utilized a microfluidics system to perform parallel real-time PCRs to test ticks for the presence of 25 bacterial and 12 parasitic species simultaneously. 

In Grenada, one of the Windward Islands of the Caribbean, *Anaplasma* and *Ehrlichia* infections are highly prevalent in dogs, and are considered endemic in cattle infections with *A. marginale* as judged by serological analysis [47,48,49,50,51,52,53], and have recently been reported in small ruminants [54]. In dogs, these infections are primarily transmitted by the vector *Rhipicephalus sanguineus* (brown dog tick). Reports on PCR and serology-based assays have identified co-infections in dogs of Grenada to both *E. canis* and *A. platys* [48,49,50]. Molecular evidence of *Anaplasma* and *Ehrlichia* infections in ruminants in Grenada, however, is limited, and infections by multiple pathogens are also not well documented [47]. Moreover, *Ehrlichia ewingii*, *Ehrlichia chaffeensis*, and *Ehrlichia ruminantium* infections have not previously been reported from Grenada, although they have been documented from some of the islands of the Caribbean [47,48,49,50,54,55]. Grenada experiences both human and animal international movements throughout the year from the Americas, Africa, Asia, and Europe since it is an educational hub and a popular tourist destination. With the population influx, the possibility of the introduction of exotic ticks and tick-borne rickettsial pathogens in Grenada is high. The currently available assays in Grenada (point-of-care enzyme-linked immunosorbent assay and conventional PCR) only focus on *E. canis* and *A. platys* due to the endemic status of these two pathogens. Due to globalization and Grenada’s unique status as a tourist and educational destination, this is no longer sufficient for the detection and control of tick-borne rickettsial infection. 

Therefore, in this study, we report the development and validation of a new multiplex PCR coupled with oligonucleotide probe based multi-analyte profiling (xMAP) bead assay for the simultaneous detection of six different *Anaplasma/Ehrlichia* species pathogens in animal-blood samples. The assay uses the basic principles of a *16S rRNA*-based real-time quantitative PCR assay, as previously described [37], combined with the Luminex xMAP hybridization technology. This technology utilizes advanced “solution-phase kinetics” in combination with optics and digital signaling to allow a high degree of multiplexing (up to 50 analytes). Other benefits of xMAP technology are the reduced sample volume requirements and fast results. The assay involves in vitro amplification of a 100 bp 16S rDNA gene fragment targeting six different species of *Anaplasma* and *Ehrlichia* and is followed by the capture and detection of species-specific amplicons using pathogen-specific complementary oligonucleotide probes attached to magnetic beads. The xMAP hybridization assay offers a distinct advantage similar to several previously reported similar methods for detecting human and veterinary pathogens [56,57,58,59]. 

## 2. Results

### 2.1. Optimization of the xMAP Assay

To develop an xMAP assay with high analytical sensitivity and specificity, optimization was performed by varying primer-annealing temperatures, amplification cycles, and varying MgCl_2_ concentrations. For the xMAP hybridization step, we optimized the probe concentration, the amount of PCR product used, hybridization time and temperature, and the ‘washed’ versus ‘no-wash’ protocols. While we used our previously reported species-specific probes for five pathogens [37], the *A. marginale*-specific probe required designing a new probe and optimization. The newly designed probe gave higher median fluorescence intensity (MFI) value for the xMAP assay when tested with the positive control plasmid. The final optimized xMAP protocol for all experiments was as follows: PCR was conducted for 35 cycles with an annealing at 50 °C for 30 s, extension at 72 °C for 30 s, and 2.5 mM of MgCl_2_. For xMAP analyses, the no-wash protocol was used with 0.1 nmols each of the probes and 5 µL of the PCR product. The probe hybridization temperature and time to achieve an optimal balance between the sensitivity and specificity were 55 °C and 15 min, respectively, for the xMAP analysis.

### 2.2. Analytical Specificity

The MFI data from all the samples in each assay was corrected for background (F − F_0_, where F is the MFI value of a sample, and F_0_ is the average background MFI value of the no-template controls (NTCs)). The multiplex analysis performed with different dilutions of positive control plasmid DNAs revealed that each species was correctly detected by its respective probe-bead set without cross-reactions with any other probe-bead sets. No positive MFI signal above the cut-off value was observed for any probe-bead set for which the corresponding specific plasmid DNA was not present. The six-plex xMAP assay had the highest (100%) analytical specificity, as no hybridization signals were observed for DNA templates from known negative animals (MFI values of negative animal samples did not differ from no-template controls) (Table 1 and Table 2). A decrease in the MFI values was observed when two plasmid combinations were present in the hybridization mix as compared to the MFI of a single species. This MFI reduction was more evident in mixtures where differences in concentrations were above one order of magnitude. For example, when 10,000 copies of *E. canis* were present singly, an average MFI value of 2088.6 was recorded (Table 1) but, when mixed with 100 copies of *E. chaffeensis*, the MFI value for *E. canis* decreased to 1500.5 (Table 2). It was also observed that some two plasmid combinations, at nonequivalent concentrations at a ratio greater than 100-fold, would only result in a positive MFI for the plasmid DNA having the higher concentration. For example, a combination of 10,000 copies of *E. chaffeensis* and 100 copies of *E. canis* gave average MFI values of 2231.6 and 28.2, respectively, without the background correction (Table 2). However, when the MFI values were corrected for background (F − F_0_), an average MFI of 2212.1, (2231.6 − 19.5) was calculated for 10,000 copies *E. chaffeensis* while 100 copies of *E. canis* resulted in an average MFI of 7.9 (28.2 − 20.3), a value below the cut-off (21.8) for *E. canis* probe-bead set (Table 2). 

### 2.3. The Limit of Detection and Analytical Sensitivity

The detection limit was 10 copies/µL for *E. canis*, *E. chaffeensis*, and *A. platys* and 100 copies/µL for *A. marginale*, *E. ewingii*, and *E. ruminantium*. For analytical sensitivity, the MFIs differed by at least two times the MFI signal between copy numbers 10, 100, 1000, and 10,000 for *E. canis*, *E. chaffeensis*, and *A. platys* species; however, for *A. marginale*, *E. ewingii*, and *E. ruminantium* the analytical sensitivity of the MFI signals was less and not able to distinguish between 10 and 100 copies of the template.

### 2.4. Repeatability

Assessment of intra-assay and inter-assay variability was determined by the percentage of coefficient of variation (%CV) of replicates run either within the plate (intra-assay) or between the plates (inter-assay). Each probe-bead set gave a different value for the intra-assay and inter-assay %CV. Therefore, the intra-assay %CV ranged between 2% to 9%, and the inter-assay %CV ranged between 4% to 19%. These values are within the acceptable range; according to Luminex [60], the values for intra-assay %CV and inter-assay %CV should be below 10 and 20, respectively.

### 2.5. Testing of the Field Samples

**Figure 1:**
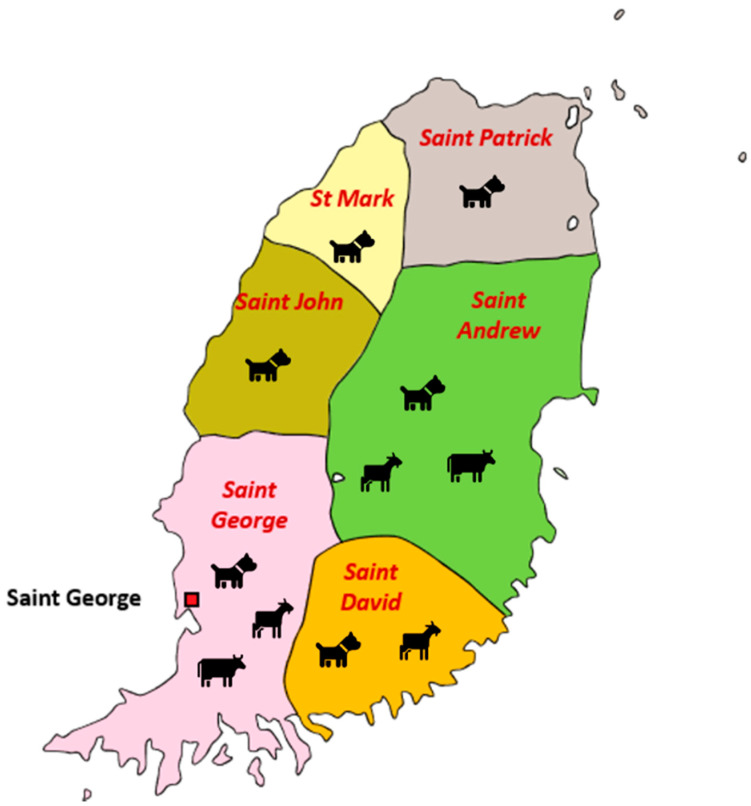
Map of Grenada showing six different parishes and the type of samples collected from each parish.

**Figure 2:**
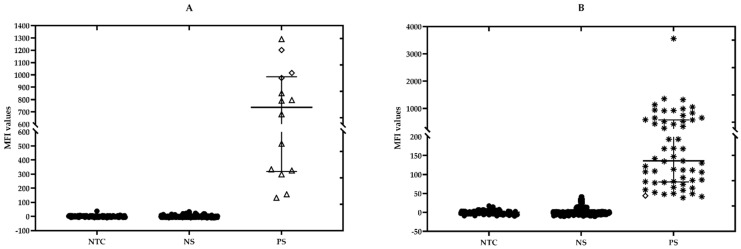

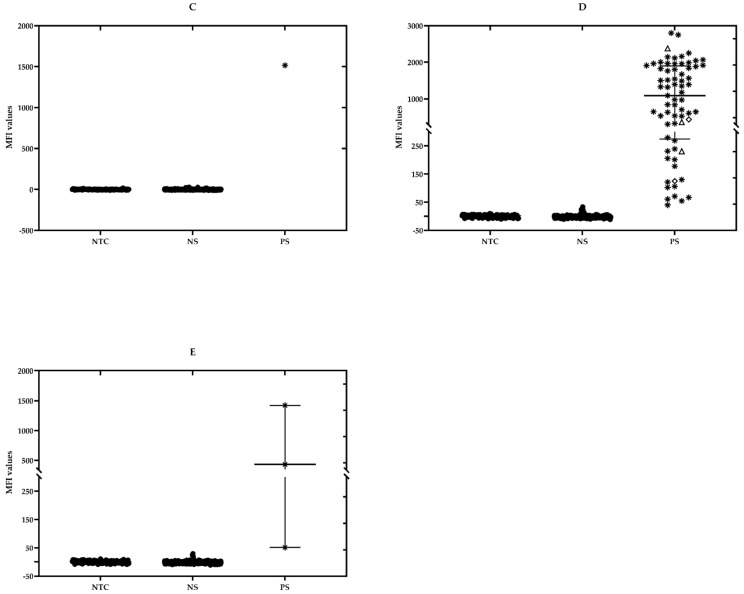
Scatter plot illustrating the distribution of corrected median fluorescent intensity values for the 455 samples obtained by the hybridization with different probes within the six-plex xMAP assay. PS: positive samples; NS: negative samples and NTC: No Template Controls (*n* = 92) from nine different assays and used to calculate the cut-offs. (**A**) *A. marginale*; (**B**) *E. canis*; (**C**) *E. ewingii*; (**D**) *A. platys*; (**E**) *E. chaffeensis*. ‘△’ blood from cattle; ‘◇’ blood from small ruminants; ‘✴’ blood from dogs. Whiskers in each plot represent the interquartile range (Median—middle line and lower and upper lines mean 25 and 75 percentiles of the distribution).

A total of 455 blood samples collected from the six parishes in Grenada were analyzed by performing PCR and xMAP assays (Table 3) (Figure 1). The geographic location with most positive samples was concentrated in the southern half of the island, in St. George and St. David’s parishes (Figure 1). Positive and negative controls were included as part of the analysis. Figure 2 represents the distribution of the MFIs for each detected bacterial species in the six-plex xMAP assay for all samples. *Anaplasma marginale* was primarily detected in cattle and small ruminant blood samples, whereas *E. canis* and *A. platys* were detected predominantly in dog blood samples. The highest MFI values detected among *A. marginale* and *A. platys* positives were 1290.3 and 2799.8, respectively. Similarly, the highest MFI values for *E. canis*, and *E. chaffeensis* positive samples were 3561.3, and 1422.8, respectively. In contrast, the lowest MFI values detected for positives were as follows: *A. marginale*, 131.8; *A. platys*, 40.2; *E. canis*, 38.7; and *E. chaffeensis*, 51.8. Sample from a dog reacted with the *E. ewingii* probe and had an MFI of 1515.8 (Figure 2). None of the samples were positive for *E. ruminantium*.

Of the 455 samples analyzed, 137 (30.1%) tested positive for one or more pathogens. Of all the positive samples, 130 (94.9%) tested positive for a single pathogen infection, and seven samples (5.1%) tested positive for infections with two different pathogens. Single-pathogen infections were the highest in cattle for *A. marginale* (11/32; 34.3%), followed by dogs for *E. canis*, *A. platys*, and *E. chaffeensis* (110/358; 30.7%). Unique findings included finding *E. canis* in one goat, and *A. platys* in the blood of five ruminants (three bovine and two small ruminants).

Co-infection was not detected in any cattle and small ruminant samples, whereas 1.9% (7/358) of the dog samples tested positive for co-infection with two rickettsial pathogens; *E. canis* and *E. chaffeensis* in two dogs, *E. canis* and *A. platys* in four dogs, and *A. platys* and *E. ewingii* in one dog (Table 4).

### 2.6. Confirmatory PCR Assays

In order to confirm and validate the results obtained from this newly developed xMAP assay, conventional PCR assays and direct sequencing were performed on the extracted genomic DNA for a subset of the field samples. The target gene for PCR assays and sequencing were *msp1a* for *A. marginale* and *16S rRNA* for *E. canis*, *E. chaffeensis*, and *E. ewingii*. The sequences shown in Table 5 had at least 94% identity with the reference sequences. All these sequences have been deposited in the GenBank (Accession numbers; MW474807-15, MW486117, and MW486118). xMAP results for *A. platys* positives were not confirmed via the conventional PCR method. 

## 3. Discussion

In this study, we described the development and application of an xMAP six-plex PCR and oligonucleotide bead-based assay having high analytical specificity and sensitivity. The three-step assay involves: 1) PCR amplification from a sample DNA targeting a 100 bp *16S rRNA* gene segment common to the six rickettsial pathogens; 2) PCR product hybridization with species-specific probes captured on magnetic beads and 3) detection of the hybrids by xMAP suspension array technology on a 96-well plate format. This assay has a quick turnaround time (3.5 h) and tests for six different pathogen DNAs simultaneously, which can be expanded to detect DNA targets from many other hemoparasite infections in a diverse host species. In particular, MagPix analyzers have the capability to test up to 84 different samples in addition to 12 controls in a 96-well plate format. Therefore, the assay has a broader applicability than a conventional PCR assay.

*Anaplasma* and *Ehrlichia* genera-specific primer sets targeting the *16S rRNA* gene fragment described previously by Sirigireddy and Ganta [37] enabled the amplification of all six selected rickettsial pathogen-specific DNAs in a single step. Within the amplicon includes variable region sequences specific for each species allowing the design of magnetic capture probes, which permitted the identification of pathogen-specific detections simultaneously on the xMAP platform. One major advantage of this assay is that it can test DNA samples from different sources as demonstrated in the present study through the application of canine, bovine, ovine, and caprine blood samples. To validate the performance characteristics of this six-plex assay, we performed experiments to define the analytical specificity and sensitivity, detection limit, and repeatability. We achieved a 100% analytical specificity for the assay after limiting the PCR cycles to 35 and optimizing the hybridization step at 55 °C. Any deviation from these two parameters resulted in either cross-reactions amongst the probe-bead sets or a decrease in the MFI signals. Although there was a decrease in the MFI signals when two different species-specific positive control plasmid DNAs were mixed at nonequivalent concentrations above 10-fold, this did not preclude the detection of the plasmids as positives. The decrease in MFI when the assay included two different DNA targets was attributed to increased competition between the amplicons during the PCR step rather than xMAP assay detection, as reported previously [61]. We observed that some two plasmid combinations, at nonequivalent concentrations at a ratio greater than 100-fold, would result in a positive MFI detection only for the pathogen DNA present at the higher concentration. This could be a potential limitation of the assay in situations where clinical samples are co-infected with two or more pathogens differing in bacteremia by greater than 100-fold. However, this issue is not likely to be clinically significant because the antibiotic treatment regime for all these pathogens is the same. The detection limit for each of the analytes was between 10 and 100 copies/µL, with good analytical sensitivity between log fold concentrations of copy numbers that were above the limit of detection. The high analytical specificity and sensitivity of this assay was not affected by spiking the samples with pathogen-negative DNA from dogs or cattle. The results from these experiments illustrate that the assay is both sensitive and specific for detecting the target species even when genomic DNA from the host species is present during PCR and xMAP analysis. 

The application of the xMAP six-plex PCR assay to 455 field samples detected 30.1% (137/455) of positives, where amongst these positive samples we found 34.3% (11/32) cattle for *A. marginale*, 16.4% (59/358) dogs for *A. platys*, and 13.9% (50/358) dogs for *E. canis*. These results are consistent with prior published reports from other endemic regions for bovine anaplasmosis and canine rickettsial pathogens of the world, including the Caribbean region [62,63,64,65,66]. Co-infection with *E. canis* and *A. platys* in the Grenadian dog population was 1.1% (4/358) and this observation is also similar to previous reports from the Caribbean islands of St. Kitts and Republic of Haiti [67,68]. In 2006, the reported prevalence of Grenadian dogs based on conventional PCR for *E. canis* and *A. platys* was 24.7% and 19.2%, respectively, with 5.5% of dual infections [50]. While the previously published data is consistent with the data reported here, the current study had a lower prevalence in *E. canis* and *A. platys*, which may reflect natural fluctuations in the pathogen distribution rather than the sensitivity differences in the assays. Although statistical comparisons were not performed between the prior published data and the current data, the reported variations in results may be due to differences in sample selection sites. Landscape and climatic differences among the various parishes in Grenada may have contributed to some variation in prevalence of the pathogens as noted in the current study compared to the previous reports. However, a more extensive study of the island is necessary. *Anaplasma marginale* has been known to infect bovine species worldwide, particularly in tropical, subtropical, and temperate regions [69,70,71,72,73]. However, in the Caribbean region, molecular detection of *A. marginale* infections in bovine species (e.g., cattle and buffalo) have only been reported from Cuba and Puerto Rico [74,75,76]. The present study augments those previous findings, and it is the first report on the molecular detection of *A. marginale* in cattle and small ruminants from Grenada. Previously, only serological data had been reported demonstrating exposure to *A. marginale* in the livestock animals in Grenada [47,53] and the current data validates the existence of the predicted cattle infections with the pathogen.

The xMAP assay analysis performed on various field specimens also revealed novel data. For example, our study is the second in reporting the presence of *E. canis* in goat blood [54]. Additional investigations are warranted to define the significance of *E. canis* infections in goats in causing disease in this host. The xMAP analysis of samples from domestic ruminants also resulted in the identification of *A. platys* DNA in five samples tested from domestic ruminants (cattle, goats, and sheep). This is the first study to report *A. platys* in these animal species. Additional investigations are necessary to determine the significance of *A. platys* infection to the ruminant population health. This study is also the first to report co-infections in dogs with *E. chaffeensis* and *E. canis* and with *E. ewingii* and *A. platys*. The sample cohort of dogs investigated in this study included some dogs having a travel history from the USA where *E. chaffeensis* and *E. ewingii* infections are more widespread. Therefore, the presence of *E. chaffeensis* and *E. ewingii* in dogs residing in Grenada may represent dogs originating from the USA. Since Grenada is a popular tourist and education destination with frequent movement of both humans and pet animals from other countries, including the USA and Canada, the introduction of new bacterial infections and tick species by means of importation of animals to the island cannot be ruled out. 

## 4. Materials and Methods

### 4.1. Collection of the Field Specimens

A total of 455 samples were collected between the years 2014 and 2018. These samples included whole blood collected from canines (*n* = 358), caprine and ovine (*n* = 65), and bovine (*n* = 32). (Table 3). The sample cohort for dogs was comprised of community-owned (mostly free roaming) dogs that were presented to the Small Animal Clinic (as part of diagnostic service) and to the Junior Surgery Laboratory (for blood sampling prior to spay and neuter surgeries) of the School of Veterinary Medicine (SVM) at St. George’s University (SGU). Cattle blood-samples were collected at farms in several parishes of Grenada (Figure 1) and from animals brought to the Large Animal Medicine and Surgery clinic of SVM, SGU. Small ruminants were sampled at various farms located in the parishes of Grenada (Figure 1). Bleeding and sample collections for all the animals were performed as per the approved Institutional Animal Care and Use Committee protocols of SGU. Blood samples were collected in EDTA-anticoagulant tubes and stored at 4 °C until processing, which typically occurred within 24 h. Table 3 represents the Grenada parish location of the blood samples collected from each animal species.

### 4.2. DNA Extraction

Genomic DNA (gDNA) from the blood samples was isolated from 100 µL of blood using the DNeasy Blood and Tissue kit (Qiagen, Hilden, Germany) as per the manufacturer’s instruction. Yield and purity of DNAs were determined using a Nanodrop™ 2000 Spectrophotometer (Thermo Scientific, Waltham, Massachusetts, US) and then all DNAs were stored at −20 °C until subsequent analyses.

### 4.3. Optimization of the PCR and xMAP Hybridization Assay Conditions

To achieve a balance between the analytical sensitivity and specificity of the assay developed in the present study, PCR and xMAP assay conditions were optimized, as shown in Table 6. Previous reports that used the PCR-based xMAP technology have reported the use of PCR cycles from 35 [77,78] up to 45 [79,80] depending on the target-genes. In the present study, *16S rRNA* gene fragment was used for the PCR protocol, and the cycling conditions were carefully optimized after testing 30, 35, and 40 cycles in combination with different primer annealing temperatures (50 °C and 52 °C) and MgCl_2_ concentrations (1.5 mM and 2.5 mM).

For the xMAP hybridization step, different concentrations of the probes (0.1 and 0.2 nmol/µL) were tested in combination with different hybridization temperatures (50, 52, 55 and 60 °C), and incubation times (10, 15, and 20 min). A washed versus a no-wash protocol was also tested [55]. The probe for *A. marginale* was redesigned since the original probe [37] performed sub-optimally with various test conditions.

### 4.4. DNA Amplification for xMAP Assay

PCRs were performed to amplify the *Anaplasma/Ehrlichia* common 100 bp fragment corresponding to the *16S rRNA* gene segment, as described previously [37] using forward primer EHRANA-F (5′-CTCAGAACGAACGCTGG-3′) and reverse primer EHRANA-R2bio (5′/5Biosg/GCATTACTCACCCGTCTGC-3′) (Integrated DNA Technologies, Coralville, Iowa, US). The reverse primer was 5′-biotinylated to allow conjugation of streptavidin phycoerythrin (SAPE) for detection via xMAP assay by Luminex (Austin, Texas, US). All amplification reactions contained 12.5 µL of 2X Platinum Hot Start Master Mix (1.5 mM MgCl_2_, 200 µM of each dNTP, and 1 U of Taq Platinum Polymerase (Invitrogen, California, US), an additional 0.5 µL of 1mM MgCl_2_ to increase the concentration to 2.5 mM, 1.25 µL of each of primers at 0.5 µM, 1 µL of gDNA template (10–20 ng/µL), and 8.5 µL of nuclease-free water to make the final volume to 25 µL. For the positive controls, six recombinant plasmid DNAs containing inserts corresponding to a 100 bp *16S rRNA* gene-segments of *A. marginale*, *A. platys*, *E. canis*, *E. chaffeensis*, *E. ewingii*, and *E. ruminantium*, as described previously [37], were diluted to the copy numbers 100, 500, 1000, and 10,000. These plasmid controls were used in the PCR assays to serve as serial dilution positive controls. Ten reactions were included at each assay to serve as NTCs where gDNA solution was replaced with nuclease-free water. PCR thermal cycler conditions consisted of an initial denaturation step of 4 min at 94 °C, followed by 35 cycles of denaturation for 30 s at 94 °C, annealing of 30 s at 50 °C and an extension of 30 s at 72 °C. Subsequently, a final extension step was set at 72 °C for 5 min and the samples were stored at 4 °C. 

### 4.5. Oligonucleotide xMAP Assay

#### 4.5.1. Oligonucleotide Probe Design

Species-specific oligonucleotide probes (size range between 21 and 32 bp) corresponding to the variable regions located within the amplicons were prepared as described previously [37]. *Anaplasma marginale*-specific probe was designed in the current study and listed in Table 7, as the previously designed probe was found to be suboptimal for the xMAP analysis. All probes were manufactured with the inclusion of a six-carbon amino linker attached to the 5′ end (Integrated DNA Technologies, Coralville, IA, USA).

#### 4.5.2. Oligonucleotide Probe Coupling to xMAP Beads

Species-specific oligonucleotide probes were conjugated to six unique sets of fluorescent-dyed magnetic carboxylated MagPlex^®^ Microspheres (beads) (Luminex, Austin, TX, USA) by a chemical reaction attaching the carboxy groups on the beads to the amine group of the 5′ end probe liners, as per the manufacturer’s protocol [60]. Six different bead-set stocks (represented as R20, R25, R34, R38, R43, R48, and R53) were then resuspended by being vortexed at 20 rpm for 1 to 2 min and sonicated for 1 min. Five million beads from each bead-set stock were coupled to amine-linked species-specific oligonucleotide probes protocol as per manufacturer’s protocol [60]. Coupling efficiency was evaluated by hybridization of the coupled beads with two-fold dilutions of femtomolar concentrations of biotinylated oligonucleotide sequences that were complementary to the probes coupled to the bead-sets. The degree of hybridization was evaluated as outlined below.

#### 4.5.3. Direct Hybridization of Blood-Derived DNA Samples to Six Oligonucleotide Probe-Coupled xMAP Beads

For these experiments, a no-wash protocol was followed [60]. Five micro liters of biotinylated PCR products were mixed with 33 µL of the six species-specific oligonucleotide bead mixtures and the volumes were raised to 50 µL with the addition of Tris-ethylene-diamine-tetraacetic acid (TE) buffer. The probe-bead mixture was calculated to contain about 23 beads/µL in 1.5× tetramethyl ammonium chloride (TMAC) hybridization buffer (4.5 M TMAC, 0.15% Sarkosyl, 75 mM Tris HCl, 6 mM EDTA pH 8.0). Biotin-labeled PCR products made from six recombinant plasmids and 10 NTCs were used as positive and negative controls, respectively. The NTCs served to calculate background MFI for each xMAP assay. The hybridization reaction was performed in Bio-Rad Hard-shell 96-well thin wall PCR plates (Hercules, CA, USA) at 55 °C for 15 min in Eppendorf Mastercycler^®^ pro (Hamburg, Germany). Twenty-five microliters of SAPE (New England Biolabs, Ipswich, MA, USA) in 1× TMAC buffer at a final concentration of 4 µg/µL was added to each reaction well and was incubated at 55 °C for a further 5 min. Each bead was analyzed by a red-light emitting diode (LED), which identified unique fluorescent dyes coating the bead region for each probe coupled bead-set and a green LED, which detects the SAPE signal of the hybridization between the amplified biotinylated-product and with complementary oligonucleotide probe(s). All analyses were performed on a MAGPIX^®^ instrument (Luminex, Austin, TX, USA) using xPONENT version 4.2 software (Luminex Corporation, Austin, TX, USA). The analysis was performed at 55 °C with an average of ~750 beads present for each of the six bead regions representing 750 replicate measurements for each bead region. An internal wash-step for each sample was carried out during the analysis to ensure removal of unbound SAPE reporter in the supernatant from interfering with the imaging chamber before reading the microspheres. The MFI data from all the samples in each assay was corrected for background (F − F_0_, where F is the MFI value of a sample, and F_0_ is the average background MFI value of the NTCs). For each probe hybridization, positive and negative cut-off values were calculated as the arithmetic mean of MFI values for the NTCs replicates included in each assay plus three standard deviations (SD) from the mean.

#### 4.5.4. Determination of the Analytical Specificity of the Luminex Assay

The analytical specificity of all probe-bead sets was tested against recombinant plasmids (i) to identify a single DNA species when all six probe-bead sets are present and (ii) to identify combinations of two different positive control plasmids when added to the six-oligo bead sets. Table 8 illustrates the experimental set-up for different plasmid combinations tested to determine the analytical specificity of the Luminex assay. Every analysis was performed with 5 µL of PCR amplicon containing positive control plasmid DNA as described in the above section. To simulate natural infection, plasmids were spiked with known negative genomic DNAs (3–5 ng/µL) recovered from dog and cattle blood. The spiked DNAs from known negatives were run separately as additional controls along with the NTCs.

#### 4.5.5. Determination of Limit of Detection and Analytical Sensitivity

Detection limit is defined as the lowest concentration of an analyte detected as positive by an assay [81]. To determine the detection limit of the assay, serial dilutions of plasmid controls of each species were used in PCRs. Six 10-fold dilutions created 10,000 to 0 copies/µL of each plasmid control [82]. To determine the ability of the assay to detect differences between MFIs of plasmid copy numbers or analytical sensitivity, the MFI of the plasmid controls were compared between each dilution or copy number. To simulate co-infections, a combination of two different control plasmids was added into wells containing the six different oligonucleotide coupled beads. All the plasmids at different concentrations were spiked with known negative dog or cattle DNA for PCR and xMAP experiments so that each reaction well also contained gDNA from negative field samples.

#### 4.5.6. Intra-Assay and Inter-Assay Variability

The intra-assay variability (repeatability or precision within a plate or run) was calculated by testing plasmid controls at concentrations of 100 and 10,000 copies/µL, in four replicates each, on a single plate. The inter-assay repeatability or precision between plates and runs was calculated by running the six plasmid controls at various dilutions (100, 500, 1000, and 10,000 copies/µL) in four different plates each run on four different days. The percent of coefficient of variation (%CV) for the intra-assay and inter-assay variability was determined by dividing the standard deviation of the replicates by the mean, then multiplied by 100.

### 4.6. Confirmation of the Results by PCR and Sequencing

Results for a subset of xMAP positive samples were confirmed using conventional PCR assays followed by sequencing. The primers targeted different genes or regions than those used for the xMAP assay (Table 9) to confirm the pathogen-DNA in the field samples. Amplicons were extracted and sent for direct sequencing to the sequencing facility of Molecular Cloning Laboratories (South San Francisco, CA, USA). The sequencing histograms were cleaned and compared to the sequence-database present in GenBank^®^ using the Nucleotide Basic Local Alignment Search Tool of the National Center for Biotechnology Information. 

## 5. Conclusions

In conclusion, this novel six-plex oligonucleotide PCR-based bead assay is highly specific, sensitive, and repeatable for the simultaneous detection of six *Anaplasma/Ehrlichia* species frequently observed in vertebrate hosts and tick vectors. The assay identified multi-infections in dogs with two *Anaplasma/Ehrlichia* species, which is consistent with prior reports in Grenada using conventional PCR. Thus, it may contribute to our understanding of the expansion of vertebrate hosts and vectors for these pathogens, their prevalence and geographic spread, and to assess possible zoonotic concerns.

## Figures and Tables

**Table 1:** Median fluorescence intensity (MFI) values of each probe-bead set shown by a single plasmid present in the hybridization mix.

DNA Targets	MFI for Hybridization of Species-Specific Oligonucleotides to DNA Targets					
	*A. marginale*	*E. canis*	*E. ewingii*	*A. platys*	*E. chaffeensis*	*E. ruminantium*
NTCs ^c^	23.5 ± 11.1 ^a^ (>34.6)	24 ± 14.1 ^a^ (>38.5)	23.1 ± 11.4 ^a^ (>35)	24.6 ± 10.8 ^a^ (>35.8)	23.1 ± 10.2 ^a^ (>33.5)	25.5 ± 10.2 ^a^ (>36)
*A. marginale ^#^*	**670.5**^b^ ± 118.6	21.6 ± 3.7	24 ± 2.6	22 ± 2.6	21.6 ± 4.6	24 ± 3.4
*E. canis ^#^*	21.6 ± 1.1	**2088.6**^b^ ± 92.5	21.6 ± 2	22 ± 2	22 ± 1	24 ± 1.7
*E. ewingii ^#^*	18.3 ± 1.5	20.3 ± 0.5	**1605.3**^b^ ± 147	20.6 ± 2.5	19.3 ± 1.1	21 ± 2
*A. platys ^#^*	21 ± 2.6	22.8 ± 3.1	21.6 ± 1.1	**1870.2**^b^ ± 270	21.3 ^b^ ± 3.2	24.6 ± 3
*E. chaffeensis ^#^*	23.5 ± 2.3	25.3 ± 2	24 ± 1.7	23.6 ± 1.1	**2715.8**^b^ ± 321.4	27 ± 1.7
*E. ruminantium ^#^*	20.3 ± 1.5	22.3 ± 2.3	21.6 ± 1.5	21 ± 1.7	19.3 ± 2	**828**^b^ ± 83.9

The MFI values shown in the table are the average MFI values of each sample run in three independent assays ± standard deviations (SD) (without background correction). ^a^ Cut-off values defined as mean ± 3SD of the no-template controls (NTCs) for each probe-bead set obtained with replicates of each sample run in three independent assays. Values in parenthesis indicates the cut off incorporating mean + 3SD. ^b^ In bold are the values considered as positive (based on mean ± 3SD). ^c^ No Template Controls (PCR grade water). ^#^ Plasmid present at 10,000 copies/µL.

**Table 2:** MFI values of each probe-bead set shown by two plasmid combinations present in the hybridization mix.

DNA Targets	MFI for Hybridization of Species-Specific Oligonucleotides to DNA Targets					
	*A. marginale*	*E. canis*	*E. ewingii*	*A. platys*	*E. chaffeensis*	*E. ruminantium*
NTC ^c^	19.2 ± 4.5 ^a^ (>23.7)	20.3 ± 1.5 ^a^ (>21.8)	20.3 ± 1.5 ^a^ (>21.8)	21.1 ± 4.2 ^a^ (>25.3)	19.5 ± 1.5 ^a^ (>21)	22 ± 2.4 ^a^ (>24.4)
Neg D ^d^	17.8 ± 0.5	19 ± 0	18 ± 0.8	19.2 ± 0.5	18 ± 0.8	20.5 ± 1
Neg C ^e^	19.5 ± 0.5	19.8 ± 0.9	19.2 ± 0.9	20 ± 0.8	19.2 ± 0.5	21.5 ± 2
EC-AP ^f^	20 ± 0	**1344.2**^b^ ± 16.2	20.5 ± 0.5	**331.1**^b^ ± 7.3	20.8 ± 1.7	23.6 ± 1.3
AP-EC ^g^	18.9 ± 0.2	**36**^b^ ± 2.1	19.8 ± 1.2	**1242.6**^b^ ± 39.3	20.2 ± 2.3	21.6 ± 1.7
EC-ECH ^h^	21.2 ± 0.9	**1500.5**^b^ ± 19.2	**22.8**^b^ ± 1.8	22 ± 0.8	**55**^b^ ± 0	**24.5**^b^ ± 1.2
ECH-EC ^i^	18.8 ± 1.7	**28.2**^b^ ± 2.6	18.5 ± 2	18.9 ± 1.6	**2231.6**^b^ ± 80.9	20.4 ± 1.7
AM-ECH ^j^	**1077.6**^b^ ± 26.8	20 ± 0.8	**25.2**^b^ ± 0.9	19.8 ± 0.5	**107.5**^b^ ± 2.6	21.8 ± 0.5
ECH-AM ^k^	**25.8**^b^ ± 1.7	**26.2**^b^ ± 1.2	**23.5**^b^ ± 0.5	24.9 ± 1	**3997**^b^ ± 120.8	**27**^b^ ± 2

The MFI values shown in the table are the average MFI values of each sample run in four replicates within the assays ± SD (without background correction). ^a^ Cut-off values defined as mean ± 3SD of the NTCs for each probe-bead set obtained with four-replicates of each sample run on the same plate. Values in parenthesis indicates the cut off incorporating mean + 3SD. ^b^ In bold are the values considered as positive (based on mean ± 3SD). ^c^ No Template Control (PCR grade water). ^d^ Spike-DNA sample from known negative dog. ^e^ Spike-DNA sample from known negative cattle. ^f^ *Ehrlichia canis* at 10,000 copies/µL mixed with *A. platys* 100 copies/µL and spiked with negative dog DNA. ^g^ *Anaplasma platys* at 10,000 copies/µL mixed with *E. canis* 100 copies/µL and spiked with negative dog DNA. ^h^ *Ehrlichia canis* at 10,000 copies/µL mixed *E. chaffeensis* 100 copies/µL and spiked with negative dog DNA. ^i^ *Ehrlichia chaffeensis* at 10,000 copies/µL mixed with *E. canis* 100 copies/µL and spiked with negative dog DNA. ^j^ *Anaplasma marginale* at 10,000 copies/µL mixed *E. chaffeensis* 100 copies/µL and spiked with negative cattle DNA. ^k^ *Ehrlichia chaffeensis* at 10,000 copies/µL mixed *A. marginale* 100 copies/µL and spiked with negative cattle DNA.

**Table 3:** The number and parish location of the animals sampled.

Species/# Sampled	# of Samples	Parish (# Sampled)							Year of Collection
		SG	SA	SM	SP	SD	SJ	Uk	
Canine/353	358 *	185	48	12	4	50	1	58	2014–2018
Caprine and Ovine/65	65	18	25	-	-	22	-	-	2017–2018
Bovine/32	32	25	7	-	-	-	-	-	2017
Total (%)	455	228 (50.1)	80 (17.6)	12 (2.6)	4 (0.8)	72 (15.8)	1 (0.2)	58 (12.7)	

* Blood samples from five dogs were collected twice (one week apart). SG: Saint George parish; SA: Saint Andrew parish; SM: Saint Mark parish; SP: Saint Patrick parish; SD: Saint David parish; SJ: Saint John parish; Uk: Unknown (location not recorded).

**Table 4:** Number and percentage of animals that tested positive for single or multiple bacterial species by the Luminex assay.

		*Bacterial* sp.	Canine	Bovine	Caprine and Ovine	Total
	*Animal* sp.					
Single speciesinfections	AM		-	11/32 (34.3)	3/65 (4.6)	14
	EC		50/358 (13.9)	**-**	**1/65 (1.5)**	51
	AP		59/358 (16.4)	**3/32 (9.3)**	**2/65 (3)**	64
	ECH		1/358 (0.2)	-	-	1
	Total		110/358 (30.7)	14/32 (43.7)	6/65 (9.2)	130
Co-infections	EC-ECH		**2/358 (0.5)**	-	-	2
	EC-AP		4/358 (1.1)	-	-	4
	EE-AP		**1/358 (0.2)**	-	-	1
	Total		7/358 (1.9)	0/36 (0)	0/65 (0)	7
	Grand Total		117/358 (32.6)	14/32 (43.7)	6/65 (9.2)	137

Unique findings are in bold. AM: *A marginale*; EC: *E. canis*; EE: *E. ewingii*; AP: *A. platys*; ECH: *E. chaffeensis*; and ER: *E. ruminantium*. Hyphenated abbreviations indicate co-infections of two different pathogens.

**Table 5:** Homology between deposited sequences and reference sequences in GenBank.

Species	Target Gene	# of Samples Tested	# of Samples Sequenced	Deposited Sequence GenBank #s	Length (bp)	Percentage of Identity (%)	Reference Sequence
*A. marginale*	*msp1a*	8	2	MW486117 MW486118	568 326	94.00 94.00	NC_012026 NC_012026
*E. canis*	*16S rRNA*	6	2	MW474807 MW474808	335 335	99.40 99.40	NR_118741 NR_118741
*E. chaffeensis*	*16S rRNA*	10	6	MW474809 MW474810 MW474811 MW474812 MW474813 MW474814	300 334 318 334 333 334	100.00 100.00 100.00 100.00 100.00 100.00	NR_074500 NR_074500 NR_074500 NR_074500 NR_074500 NR_074500
*E. ewingii*	*16S rRNA*	6	1	MW474815	308	100.00	NR_074500

**Table 6:** xMAP assay optimization at different levels.

	Optimization Conditions	Test Conditions
PCR optimization	Primer Annealing temperature (°C)	50, 52
	MgCl_2_ concentration (mM)	1.5, 2.5
	PCR cycle numbers	30, 35, 40
xMAP optimization	Hybridization Temperature (°C)	50, 52, 55, 60
	Hybridization time (min.)	10, 15, 20
	PCR product volume (µL)	5, 10
	xMAP protocol	Washed, no-wash
	Concentration of the probes (nmol/µL)	0.1, 0.2

**Table 7:** Sequences of the oligonucleotide probes that were covalently linked to the carboxylated microspheres used in the development of xMAP assay for the detection of species identification of *Ehrlichia/Anaplasma* in animals.

Probes	Bacterial Species	Sequences (5′-3′)	xMAP COOH-Microsphere Regions for Probe Binding	Reference
RG270Ecan	*E. canis*	TATAGCCTCTGGCTATAGGAAATTGTTAG	R25	[37]
RG266Echaf	*E. chaffeensis*	CTTATAACCTTTTGGTTATAAATAATTGTTAG	R43	[37]
RG268Eewin	*E. ewingii*	CTAAATAGTCTCTGATTTAGATAGTTGTTAG	R34	[37]
RG260Erum	*E. ruminantium*	GTTATTTATAGCTTCGGCTAT	R48	[37]
RG272Aplat	*A. platys*	CGGATTTTTGTCGTAGCTTGCTATGAT	R38	[37]
RG262Amarg	*A. marginale*	CGTATACGCAGCTTGCTGCGT	R20	This study

R20, R25, R34, R38, R43, and R48 represent bead-set stocks with different spectral properties.

**Table 8:** Combinations of plasmid DNA mixes analyzed to determine analytical specificity.

Species		Mix							
	1 ^✴^	2 ^✴^	3 ^✴^	4 ^✴^	5 ^✴^	6 ^✴^	7 and 8 ^★^	9 and 10 ^★^	11 and 12 ^★^
*E. canis*	✓	✗	✗	✗	✗	✗	✓	✓	✗
*E. chaffeensis*	✗	✓	✗	✗	✗	✗	✗	✓	✓
*E. ewingii*	✗	✗	✓	✗	✗	✗	✗	✗	✗
*E. ruminantium*	✗	✗	✗	✓	✗	✗	✗	✗	✗
*A. platys*	✗	✗	✗	✗	✓	✗	✓	✗	✗
*A. marginale*	✗	✗	✗	✗	✗	✓	✗	✗	✓

✓ Shows the presence of the corresponding bacterial plasmid DNA in the mix. ✗ Indicates the absence of the corresponding bacterial plasmid DNA in the mix. ^✴^ Positive control plasmid mixtures 1 to 6 contain single bacterial species (indicated by ✓) at 10,000 copies/µL in each mixture. ^★^ Positive control plasmid mixtures 7 and 8, 9 and 10, and 11 and 12 contain two bacterial species (indicated by ✓) at 10,000 and 100 copies/µL.

**Table 9:** Primers used for confirmatory PCR assays for various species.

Species	Target Gene	Primer Name	Sequence (5′→3′)	Amplicon Size (bp)	Reference
*A. marginale*	*msp1a*	MSP1aF1 MSP1aRN	GCATTACAACGCAACGCTTGAG CAGGAGCACCACCAAACATCATCACA	1638	This study
*A. platys*	*16S rRNA*	EP2 EP3	GAAGATAATGACGGTACCC CGTTTTGTCTCTGTGTTG	385	[83]
*E. canis*	*16S rRNA*	ECA HE3	CAATTATTTATAGCCTCTGGCTATAGG TATAGGTACCGTCATTATCTTCCCTAT	385	[34]
*E. chaffeensis*	*16S rRNA*	HE1 HE3	CAATTGCTTATAACCTTTTGGTTATAAAT TATAGGTACCGTCATTATCTTCCCTAT	385	[84]
*E. ewingii*	*16S rRNA*	EE72 HE3	CAATTCCTAAATAGTCTCTGACTATT TATAGGTACCGTCATTATCTTCCCTAT	385	[85]

## Data Availability

The new nucleic acid sequences have been deposited in the database of GenBank. Accession numbers provided by GenBank have been included in the manuscript under the Table 5.

## References

[1] Dumler J.S., Barbet A.F., Bekker C.P., Dasch G.A., Palmer G.H., Ray S.C., Rikihisa Y., Rurangirwa F.R. (2001). Reorganization of genera in the families Rickettsiaceae and Anaplasmataceae in the order Rickettsiales: Unification of some species of Ehrlichia with Anaplasma, Cowdria with Ehrlichia and Ehrlichia with Neorickettsia, descriptions of six new species combinations and designation of Ehrlichia equi and ‘HGE agent’ as subjective synonyms of Ehrlichia phagocytophila. Int. J. Syst. Evol. Microbiol..

[2] Rymaszewska A., Grenda S. (2008). Bacteria of the genus Anaplasma–characteristics of Anaplasma and their vectors: A review. Vet. Med..

[3] Rar V., Golovljova I. (2011). Anaplasma, Ehrlichia, and “Candidatus Neoehrlichia” bacteria: Pathogenicity, biodiversity, and molecular genetic characteristics, a review. Infect. Genet. Evol..

[4] Centers for Disease Control and Prevention Tickborne Disease Surveillance Data Summary. https://www.cdc.gov/ticks/data-summary/index.html.

[5] Alhassan A., Pumidonming W., Okamura M., Hirata H., Battsetseg B., Fujisaki K., Yokoyama N., Igarashi I. (2005). Development of a single-round and multiplex PCR method for the simultaneous detection of Babesia caballi and Babesia equi in horse blood. Vet. Parasitol..

[6] Breitschwerdt E.B., Hegarty B.C., Hancock S.I. (1998). Sequential evaluation of dogs naturally infected with Ehrlichia canis, Ehrlichia chaffeensis, Ehrlichia equi, Ehrlichia ewingii, or Bartonella vinsonii. J. Clin. Microbiol..

[7] Chang Y.F., Novosel V., Chang C.F., Kim J.B., Shin S.J., Lein D.H. (1998). Detection of human granulocytic ehrlichiosis agent and Borrelia burgdorferi in ticks by polymerase chain reaction. J. Vet. Diagn. Investig..

[8] Cui Y., Zhang Y., Jian F., Zhang L., Wang R., Cao S., Wang X., Yan Y., Ning C. (2017). Development of duplex PCR for simultaneous detection of Theileria spp. and Anaplasma spp. in sheep and goats. Exp. Parasitol..

[9] Hoskins J.D., Breitschwerdt E.B., Gaunt S.D., French T.W., Burgdorfer W. (1988). Antibodies to Ehrlichia canis, Ehrlichia platys, and spotted fever group rickettsiae in Louisiana dogs. J. Vet. Intern. Med..

[10] Hua P., Yuhai M., Shide T., Yang S., Bohai W., Xiangrui C. (2000). Canine ehrlichiosis caused simultaneously by Ehrlichia canis and Ehrlichia platys. Microbiol. Immunol..

[11] Kordick S.K., Breitschwerdt E.B., Hegarty B.C., Southwick K.L., Colitz C.M., Hancock S.I., Bradley J.M., Rumbough R., McPherson J.T., MacCormack J.N. (1999). Coinfection with multiple tick-borne pathogens in a Walker Hound kennel in North Carolina. J. Clin. Microbiol..

[12] Lorusso V., Wijnveld M., Majekodunmi A.O., Dongkum C., Fajinmi A., Dogo A.G., Thrusfield M., Mugenyi A., Vaumourin E., Igweh A.C. (2016). Tick-borne pathogens of zoonotic and veterinary importance in Nigerian cattle. Parasites Vectors.

[13] Maggi R.G., Mascarelli P.E., Havenga L.N., Naidoo V., Breitschwerdt E.B. (2013). Co-infection with Anaplasma platys, Bartonella henselae and Candidatus Mycoplasma haematoparvum in a veterinarian. Parasit Vectors.

[14] Meinkoth J.H., Ewing S.A., Cowell R.L., Dawson J.E., Warner C.K., Mathew J.S., Bowles M., Thiessen A.E., Panciera R.J., Fox C. (1998). Morphologic and molecular evidence of a dual species ehrlichial infection in a dog presenting with inflammatory central nervous system disease. J. Vet. Intern. Med..

[15] Njiiri N.E., Bronsvoort B.M., Collins N.E., Steyn H.C., Troskie M., Vorster I., Thumbi S.M., Sibeko K.P., Jennings A., van Wyk I.C. (2015). The epidemiology of tick-borne haemoparasites as determined by the reverse line blot hybridization assay in an intensively studied cohort of calves in western Kenya. Vet. Parasitol..

[16] Rajput Z.I., Hu S.H., Arijo A.G., Habib M., Khalid M. (2005). Comparative study of Anaplasma parasites in tick carrying buffaloes and cattle. J. Zhejiang Univ. Sci. B.

[17] Ringo A.E., Adjou Moumouni P.F., Taioe M., Jirapattharasate C., Liu M., Wang G., Gao Y., Guo H., Lee S.H., Zheng W. (2018). Molecular analysis of tick-borne protozoan and rickettsial pathogens in small ruminants from two South African provinces. Parasitol. Int..

[18] De Tommasi A.S., Otranto D., Dantas-Torres F., Capelli G., Breitschwerdt E.B., de Caprariis D. (2013). Are vector-borne pathogen co-infections complicating the clinical presentation in dogs?. Parasites Vectors.

[19] Mylonakis M.E., Koutinas A.F., Baneth G., Polizopoulou Z., Fytianou A. (2004). Mixed Ehrlichia canis, Hepatozoon canis, and presumptive Anaplasma phagocytophilum infection in a dog. Vet. Clin. Pathol..

[20] Tuttle A.D., Birkenheuer A.J., Juopperi T., Levy M.G., Breitschwerdt E.B. (2003). Concurrent bartonellosis and babesiosis in a dog with persistent thrombocytopenia. J. Am. Vet. Med. Assoc..

[21] Gaunt S., Beall M., Stillman B., Lorentzen L., Diniz P., Chandrashekar R., Breitschwerdt E. (2010). Experimental infection and co-infection of dogs with Anaplasma platys and Ehrlichia canis: Hematologic, serologic and molecular findings. Parasit Vectors.

[22] Gal A., Harrus S., Arcoh I., Lavy E., Aizenberg I., Mekuzas-Yisaschar Y., Baneth G. (2007). Coinfection with multiple tick-borne and intestinal parasites in a 6-week-old dog. Can. Vet. J..

[23] Otranto D., Dantas-Torres F., Breitschwerdt E.B. (2009). Managing canine vector-borne diseases of zoonotic concern: Part two. Trends Parasitol..

[24] Chapman A.S., Bakken J.S., Folk S.M., Paddock C.D., Bloch K.C., Krusell A., Sexton D.J., Buckingham S.C., Marshall G.S., Storch G.A. (2006). Diagnosis and management of tickborne rickettsial diseases: Rocky Mountain spotted fever, ehrlichioses, and anaplasmosis--United States: A practical guide for physicians and other health-care and public health professionals. MMWR Recomm. Rep..

[25] Reller M.E., Dumler J.S. (2015). Ehrlichia, Anaplasma, and related intracellular bacteria. Man. Clin. Microbiol..

[26] Walker D.H., Bouyer D.H., Jorgensen J.H., Carroll K.C., Funke G., Pfaller M.A., Landry M.L., Richter S.S., Warnock D.W., Carroll K.C., Funke G., Bernard K.A. (2015). Rickettsia and orientia. Manual of Clinical Microbiology.

[27] Chandrashekar R., Mainville C.A., Beall M.J., O’Connor T., Eberts M.D., Alleman A.R., Gaunt S.D., Breitschwerdt E.B. (2010). Performance of a commercially available in-clinic ELISA for the detection of antibodies against Anaplasma phagocytophilum, Ehrlichia canis, and Borrelia burgdorferi and Dirofilaria immitis antigen in dogs. Am. J. Vet. Res..

[28] Malheiros J., Costa M.M., do Amaral R.B., de Sousa K.C.M., André M.R., Machado R.Z., Vieira M.I.B. (2016). Identification of vector-borne pathogens in dogs and cats from Southern Brazil. Ticks Tick Borne Dis..

[29] O’Connor T.P., Hanscom J.L., Hegarty B.C., Groat R.G., Breitschwerdt E.B. (2006). Comparison of an indirect immunofluorescence assay, western blot analysis, and a commercially available ELISA for detection of Ehrlichia canis antibodies in canine sera. Am. J. Vet. Res..

[30] Biggs H.M., Behravesh C.B., Bradley K.K., Dahlgren F.S., Drexler N.A., Dumler J.S., Folk S.M., Kato C.Y., Lash R.R., Levin M.L. (2016). Diagnosis and Management of Tickborne Rickettsial Diseases: Rocky Mountain Spotted Fever and Other Spotted Fever Group Rickettsioses, Ehrlichioses, and Anaplasmosis—United States. MMWR Recomm. Rep..

[31] World Organization for Animal Health Manual of Diagnostic Tests and Vaccines for Terrestrial Animals, Chapter 3.4.1. Bovine Anaplasmosis. https://www.oie.int/en/standard-setting/terrestrial-manual/access-online/.

[32] Chen S.M., Dumler J.S., Bakken J.S., Walker D.H. (1994). Identification of a granulocytotropic Ehrlichia species as the etiologic agent of human disease. J. Clin. Microbiol..

[33] Courtney J.W., Kostelnik L.M., Zeidner N.S., Massung R.F. (2004). Multiplex real-time PCR for detection of anaplasma phagocytophilum and Borrelia burgdorferi. J. Clin. Microbiol..

[34] Dawson J.E., Biggie K.L., Warner C.K., Cookson K., Jenkins S., Levine J.F., Olson J.G. (1996). Polymerase chain reaction evidence of Ehrlichia chaffeensis, an etiologic agent of human ehrlichiosis, in dogs from southeast Virginia. Am. J. Vet. Res..

[35] Eddlestone S.M., Gaunt S.D., Neer T.M., Boudreaux C.M., Gill A., Haschke E., Corstvet R.E. (2007). PCR detection of Anaplasma platys in blood and tissue of dogs during acute phase of experimental infection. Exp. Parasitol..

[36] Hulínská D., Langrová K., Pejcoch M., Pavlásek I. (2004). Detection of Anaplasma phagocytophilum in animals by real-time polymerase chain reaction. Apmis.

[37] Sirigireddy K.R., Ganta R.R. (2005). Multiplex detection of Ehrlichia and Anaplasma species pathogens in peripheral blood by real-time reverse transcriptase-polymerase chain reaction. J. Mol. Diagn..

[38] Doyle C.K., Labruna M.B., Breitschwerdt E.B., Tang Y.W., Corstvet R.E., Hegarty B.C., Bloch K.C., Li P., Walker D.H., McBride J.W. (2005). Detection of medically important Ehrlichia by quantitative multicolor TaqMan real-time polymerase chain reaction of the dsb gene. J. Mol. Diagn..

[39] Benevenute J.L., Dumler J.S., Ogrzewalska M., Roque A.L.R., Mello V.V.C., de Sousa K.C.M., Gonçalves L.R., D’Andrea P.S., de Sampaio Lemos E.R., Machado R.Z. (2017). Assessment of a quantitative 5’ nuclease real-time polymerase chain reaction using groEL gene for Ehrlichia and Anaplasma species in rodents in Brazil. Ticks Tick Borne Dis..

[40] Lew A.E., Gale K.R., Minchin C.M., Shkap V., de Waal D.T. (2003). Phylogenetic analysis of the erythrocytic Anaplasma species based on 16S rDNA and GroEL (HSP60) sequences of A. marginale, A. centrale, and A. ovis and the specific detection of A. centrale vaccine strain. Vet. Microbiol..

[41] Lew A.E., Bock R.E., Minchin C.M., Masaka S. (2002). A msp1alpha polymerase chain reaction assay for specific detection and differentiation of Anaplasma marginale isolates. Vet. Microbiol..

[42] Vidotto M.C., Kano S.F., Gregori F., Headley S.A., Vidotto O. (2006). Phylogenetic analysis of Anaplasma marginale strains from Paraná State, Brazil, using the msp1alpha and msp4 genes. J. Vet. Med. B Infect. Dis. Vet. Public Health.

[43] Gusa A.A., Buller R.S., Storch G.A., Huycke M.M., Machado L.J., Slater L.N., Stockham S.L., Massung R.F. (2001). Identification of a p28 gene in Ehrlichia ewingii: Evaluation of gene for use as a target for a species-specific PCR diagnostic assay. J. Clin. Microbiol..

[44] Zhang C., Xiong Q., Kikuchi T., Rikihisa Y. (2008). Identification of 19 polymorphic major outer membrane protein genes and their immunogenic peptides in Ehrlichia ewingii for use in a serodiagnostic assay. Clin. Vaccine Immunol..

[45] Inokuma H., Brouqui P., Drancourt M., Raoult D. (2001). Citrate synthase gene sequence: A new tool for phylogenetic analysis and identification of Ehrlichia. J. Clin. Microbiol..

[46] Michelet L., Delannoy S., Devillers E., Umhang G., Aspan A., Juremalm M., Chirico J., van der Wal F.J., Sprong H., Boye Pihl T.P. (2014). High-throughput screening of tick-borne pathogens in Europe. Front. Cell. Infect. Microbiol..

[47] Gibson K., Fitzpatrick D., Stone D., Noel T., MacPherson C. (2016). Vector-borne diseases in the Caribbean: History and current status. Cab. Rev..

[48] Lanza-Perea M., Zieger U., Qurollo B.A., Hegarty B.C., Pultorak E.L., Kumthekar S., Bruhl-Day R., Breitschwerdt E.B. (2014). Intraoperative bleeding in dogs from Grenada seroreactive to Anaplasma platys and Ehrlichia canis. J. Vet. Intern. Med..

[49] Wilkerson M.J., Black K.E., Lanza-Perea M., Sharma B., Gibson K., Stone D.M., George A., Nair A.D., Ganta R.R. (2017). Initial development and preliminary evaluation of a multiplex bead assay to detect antibodies to Ehrlichia canis, Anaplasma platys, and Ehrlichia chaffeensis outer membrane peptides in naturally infected dogs from Grenada, West Indies. J. Vet. Diagn. Investig..

[50] Yabsley M.J., McKibben J., Macpherson C.N., Cattan P.F., Cherry N.A., Hegarty B.C., Breitschwerdt E.B., O’Connor T., Chandrashekar R., Paterson T. (2008). Prevalence of Ehrlichia canis, Anaplasma platys, Babesia canis vogeli, Hepatozoon canis, Bartonella vinsonii berkhoffii, and Rickettsia spp. in dogs from Grenada. Vet. Parasitol..

[51] Camus E., Barré N. (1995). Vector situation of tick-borne diseases in the Caribbean islands. Vet. Parasitol..

[52] Camus E., Maran M., Montenegro-James S., Accipe A. (1998). Sero-Epidemiological Survey on Bovine Tick-Borne Diseases in the Lesser Antilles. Proceedings of the Final Research Co-Ordination Meetings of FAO/IAEA/SIDA Co-Ordinated Research Projects.

[53] Camus E., Montenegro-James S. (1994). Bovine anaplasmosis and babesiosis in the Lesser Antilles: Risk assessment of an unstable epidemiologic situation. Vet. Res..

[54] Zhang J., Kelly P., Guo W., Xu C., Wei L., Jongejan F., Loftis A., Wang C. (2015). Development of a generic Ehrlichia FRET-qPCR and investigation of ehrlichioses in domestic ruminants on five Caribbean islands. Parasit. Vectors.

[55] Gondard M., Cabezas-Cruz A., Charles R.A., Vayssier-Taussat M., Albina E., Moutailler S. (2017). Ticks and Tick-Borne Pathogens of the Caribbean: Current Understanding and Future Directions for More Comprehensive Surveillance. Front. Cell. Infect. Microbiol..

[56] Christopher-Hennings J., Araujo K.P., Souza C.J., Fang Y., Lawson S., Nelson E.A., Clement T., Dunn M., Lunney J.K. (2013). Opportunities for bead-based multiplex assays in veterinary diagnostic laboratories. J. Vet. Diagn. Investig..

[57] Livengood J., Hutchinson M.L., Thirumalapura N., Tewari D. (2020). Detection of Babesia, Borrelia, Anaplasma, and Rickettsia spp. in Adult Black-Legged Ticks (Ixodes scapularis) from Pennsylvania, United States, with a Luminex Multiplex Bead Assay. Vector Borne Zoonotic Dis..

[58] Reslova N., Huvarova V., Hrdy J., Kasny M., Kralik P. (2019). A novel perspective on MOL-PCR optimization and MAGPIX analysis of in-house multiplex foodborne pathogens detection assay. Sci. Rep..

[59] Reslova N., Michna V., Kasny M., Mikel P., Kralik P. (2017). xMAP Technology: Applications in Detection of Pathogens. Front. Microbiol..

[60] Angeloni S.D.S., Dunbar S., Stone V., Swift S. xMAP® Cookbook. A Collection of Methods and Protocols for Developing Multiplex Assays with xMAP Technology. https://cdn2.hubspot.net/hubfs/128032/Cookbook/BR76862.xMAPCookbook.Ed4.WR.pdf.

[61] Ros-García A., Juste R.A., Hurtado A. (2012). A highly sensitive DNA bead-based suspension array for the detection and species identification of bovine piroplasms. Int. J. Parasitol..

[62] Abanda B., Paguem A., Abdoulmoumini M., Kingsley M.T., Renz A., Eisenbarth A. (2019). Molecular identification and prevalence of tick-borne pathogens in zebu and taurine cattle in North Cameroon. Parasit. Vectors.

[63] Kelly P.J., Xu C., Lucas H., Loftis A., Abete J., Zeoli F., Stevens A., Jaegersen K., Ackerson K., Gessner A. (2013). Ehrlichiosis, babesiosis, anaplasmosis and hepatozoonosis in dogs from St. Kitts, West Indies. PLoS ONE.

[64] Lara B., Conan A., Thrall M.A., Ketzis J.K., Branford G.C., Rajeev S. (2020). Serologic and Molecular Diagnosis of Anaplasma platys and Ehrlichia canis Infection in Dogs in an Endemic Region. Pathogens.

[65] Peter S.G., Aboge G.O., Kariuki H.W., Kanduma E.G., Gakuya D.W., Maingi N., Mulei C.M., Mainga A.O. (2020). Molecular prevalence of emerging Anaplasma and Ehrlichia pathogens in apparently healthy dairy cattle in peri-urban Nairobi, Kenya. BMC Vet. Res..

[66] Tana-Hernández L., Navarrete-Arroyo K., Ron-Román J., Reyna-Bello A., Chávez-Larrea M.A. (2017). PCR-diagnosis of Anaplasma marginale in cattle populations of Ecuador and its molecular identification through sequencing of ribosomal 16S fragments. BMC Vet. Res..

[67] Loftis A.D., Kelly P.J., Freeman M.D., Fitzharris S., Beeler-Marfisi J., Wang C. (2013). Tick-borne pathogens and disease in dogs on St. Kitts, West Indies. Vet. Parasitol..

[68] Starkey L.A., Newton K., Brunker J., Crowdis K., Edourad E.J.P., Meneus P., Little S.E. (2016). Prevalence of vector-borne pathogens in dogs from Haiti. Vet. Parasitol..

[69] Carelli G., Decaro N., Lorusso A., Elia G., Lorusso E., Mari V., Ceci L., Buonavoglia C. (2007). Detection and quantification of Anaplasma marginale DNA in blood samples of cattle by real-time PCR. Vet. Microbiol..

[70] Cossío-Bayúgar R., Rodríguez S.D., García-Ortiz M.A., García-Tapia D., Aboytes-Torres R. (1997). Bovine anaplasmosis prevalence in northern Veracruz state, Mexico. Prev. Vet. Med..

[71] da Silva N.B., Taus N.S., Johnson W.C., Mira A., Schnittger L., Valente J.D.M., Vidotto O., Masterson H.E., Vieira T., Ueti M.W. (2018). First report of Anaplasma marginale infection in goats, Brazil. PLoS ONE.

[72] Torioni de Echaide S., Knowles D.P., McGuire T.C., Palmer G.H., Suarez C.E., McElwain T.F. (1998). Detection of cattle naturally infected with Anaplasma marginale in a region of endemicity by nested PCR and a competitive enzyme-linked immunosorbent assay using recombinant major surface protein 5. J. Clin. Microbiol..

[73] Yousefi A., Rahbari S., Shayan P., Sadeghi-dehkordi Z., Bahonar A. (2017). Molecular detection of Anaplasma marginale and Anaplasma ovis in sheep and goat in west highland pasture of Iran. Asian Pac. J. Trop. Biomed..

[74] Fosgate G.T., Urdaz-Rodríguez J.H., Dunbar M.D., Rae D.O., Donovan G.A., Melendez P., Dobek G.L., Alleman A.R. (2010). Diagnostic accuracy of methods for detecting Anaplasma marginale infection in lactating dairy cattle of Puerto Rico. J. Vet. Diagn. Investig..

[75] Díaz-Sánchez A.A., Meli M.L., Obregón Álvarez D., Fonseca-Rodríguez O., Cabezas-Cruz A., Hofmann-Lehmann R., Corona-González B. (2020). Development and application of a multiplex TaqMan® real-time qPCR assay for the simultaneous detection of Anaplasma marginale and Theileria annulata and molecular characterization of Anaplasma marginale from cattle in Western Cuba. Ticks Tick Borne Dis..

[76] Obregón D., Cabezas-Cruz A., Armas Y., Silva J.B., Fonseca A.H., André M.R., Alfonso P., Oliveira M.C.S., Machado R.Z., Corona-González B. (2019). High co-infection rates of Babesia bovis, Babesia bigemina, and Anaplasma marginale in water buffalo in Western Cuba. Parasitol. Res..

[77] Bergval I., Sengstake S., Brankova N., Levterova V., Abadía E., Tadumaze N., Bablishvili N., Akhalaia M., Tuin K., Schuitema A. (2012). Combined species identification, genotyping, and drug resistance detection of Mycobacterium tuberculosis cultures by MLPA on a bead-based array. PLoS ONE.

[78] Wuyts V., Roosens N.H., Bertrand S., Marchal K., De Keersmaecker S.C. (2015). Guidelines for optimisation of a multiplex oligonucleotide ligation-PCR for characterisation of microbial pathogens in a microsphere suspension array. BioMed Res. Int..

[79] Deshpande A., Gans J., Graves S.W., Green L., Taylor L., Kim H.B., Kunde Y.A., Leonard P.M., Li P.E., Mark J. (2010). A rapid multiplex assay for nucleic acid-based diagnostics. J. Microbiol. Methods.

[80] Thierry S., Hamidjaja R.A., Girault G., Löfström C., Ruuls R., Sylviane D. (2013). A multiplex bead-based suspension array assay for interrogation of phylogenetically informative single nucleotide polymorphisms for Bacillus anthracis. J. Microbiol. Methods.

[81] Armbruster D.A., Pry T. (2008). Limit of blank, limit of detection and limit of quantitation. Clin. Biochem. Rev..

[82] Sharma B. (2020). Development of a PCR Based Direct DNA Hybridization Oligonucleotide Microbead Assay for Detection of Ehrlichia and Anaplasma Species in Animals and Ticks from Grenada, West Indies. Ph.D. Thesis.

[83] Chang W.L., Pan M.J. (1996). Specific amplification of Ehrlichia platys DNA from blood specimens by two-step PCR. J. Clin. Microbiol..

[84] Anderson B.E., Sumner J.W., Dawson J.E., Tzianabos T., Greene C.R., Olson J.G., Fishbein D.B., Olsen-Rasmussen M., Holloway B.P., George E.H. (1992). Detection of the etiologic agent of human ehrlichiosis by polymerase chain reaction. J. Clin. Microbiol..

[85] Anderson B.E., Greene C.E., Jones D.C., Dawson J.E. (1992). Ehrlichia ewingii sp. nov., the etiologic agent of canine granulocytic ehrlichiosis. Int. J. Syst. Bacteriol..

